# Optimization and Standardization of Thermal Treatment as a Plasma Prefractionation Method for Proteomic Analysis

**DOI:** 10.1155/2019/8646039

**Published:** 2019-04-30

**Authors:** Wararat Chiangjong, Channarong Changtong, Jirawan Panachan, Churat Weeraphan, Chantragan Srisomsap, Suradej Hongeng, Jisnuson Svasti, Somchai Chutipongtanate

**Affiliations:** ^1^Pediatric Translational Research Unit, Department of Pediatrics, Faculty of Medicine Ramathibodi Hospital, Mahidol University, Bangkok, Thailand; ^2^Hematology and Oncology Division, Department of Pediatrics, Faculty of Medicine Ramathibodi Hospital, Mahidol University, Bangkok, Thailand; ^3^Department of Molecular Biology and Bioinformatics, Faculty of Science, Prince of Songkla University, Songkla, Thailand; ^4^Laboratory of Biochemistry, Chulabhorn Research Institute, Bangkok, Thailand; ^5^Applied Biological Sciences Program, Chulabhorn Graduate Institute, Bangkok, Thailand

## Abstract

Prefractionation is a prerequisite step for deep plasma proteomics. Highly abundant proteins, particularly human serum albumin (HSA) and immunoglobulin G (IgG), typically interfere with investigation of proteins with lower abundance. A relatively simple preparation method based on high temperature can precipitate thermolabile proteins, providing a strategic window to access the thermostable plasma subproteome. This study aimed to optimize thermal treatment as a reliable prefractionation method and to compare it with two commercial kits, including HSA and IgG immunodepletion (IMDP) and combinatorial peptide ligand libraries (CPLL), using untreated plasma as a control condition. By varying the temperature and the incubation period, the optimal condition was found as treatment at 95°C for 20 min, which maintained about 1% recovery yield of soluble proteins. Consistency and reproducibility of thermal treatment-derived plasma subproteome were checked by two-dimensional electrophoresis. The coefficient of variation regarding protein spot numbers was less than 10% among three independent specimens. Highly abundant protein depletion of the thermal treatment was evaluated by immunoblotting against HSA and IgG as compared to the untreated plasma, IMDP, and CPLL. Multidimensional comparison based on 489 unique peptides derived from the label-free quantitative mass spectrometry revealed that the thermal treatment, IMDP, and CPLL provided distinct sets of plasma subproteome compared to untreated plasma, and these appeared to be complementary to each other. Comparing the characteristics of the three procedures suggested that thermal treatment was more cost-effective and less time-consuming than IMDP and CPLL. This study proposes the use of thermal treatment as a reliable and cost-effective method for plasma prefractionation which provides benefits to large-scale proteomic projects and biomarker studies.

## 1. Introduction

Plasma is an important biological sample for clinical investigations and biomedical research. Plasma is relatively easy to access and can show significant changes in biological markers, which often relate to pathological conditions. However, the broad dynamic range of plasma proteins (>10 orders of magnitude) and the overwhelming presence of high abundant proteins particularly human serum albumin (HSA) and immunoglobulin G (IgG), which constitute more than 60-70% of whole plasma proteins, represent challenges for plasma proteomics [1, 2]. Plasma prefractionation is therefore a prerequisite step to reduce the plasma protein complexity and increase the chance of discovering clinical-relevant biomarkers. General approaches in plasma prefractionation include immunodepletion, affinity enrichment, and fractionation [3]. Choosing the proper prefractionation method can improve the outcome of plasma proteomic projects [3].

To date, the standard prefractionation methods such as HSA and IgG immunodepletion (IMDP) and combinatorial peptide ligand libraries (CPLL) are commercially available and very effective. However, disadvantages such as small sample loading capacity (which affects downstream analyses), the complexity of the procedure (which reduces sample throughput and productivity), or high unit cost (which burdens large-scale studies) are of concern. In this context, development of a prefractionation method which is reproducible, time-saving, and cost-effective would be beneficial to large-scale proteomic studies and for the future development of clinical proteomic assays [3].

Thermal treatment has been used in biomedical research [5–10], and once applied in proteomic studies [11, 12]. Thermal treatment separates plasma proteins based on their physical properties under high temperature into two fractions, namely, a thermostable (TS) protein-soluble fraction and a thermolabile protein-precipitate [3]. This process is also known as heat-induced gelation of plasma proteins [13, 14]. Fourier transform Raman spectroscopy showed changes in the secondary structures of albumin and globulins, i.e., reduced *α*-helix, disulfide bond interactions, aberrant exposure, and buriedness of hydrophobic residues, together with formation of *β*-sheet induced by fibrinogens, mainly contributing to this heat-induced gelation process of plasma [15]. Thermal treatment has several characteristics useful as a prefractionation method for large-scale proteomic studies, since it has high sample loading capacity, is simple to perform with relatively low cost, and can likely be readily automated. However, this fractionation method has not been well standardized or shown to provide highly reproducible results. These concerns need to be addressed to demonstrate the value of thermal prefractionation for application in plasma proteomic projects.

This study aimed to optimize and standardize the thermal treatment for plasma prefractionation by varying the temperature and incubation period, measuring the recovery yield, and evaluating the reproducibility of TS plasma subproteome by two-dimensional electrophoresis (2-DE). The optimized thermal treatment was then compared against two standard methods, i.e., IMDP and CPLL, using Western blot analysis and label-free quantitative mass spectrometry, where the untreated plasma served as the control condition. Finally, the characteristics of the thermal treatment, IMDP, and CPLL procedures were compared. This study provided evidence to support future application of thermal treatment in large-scale plasma proteomic projects.

## 2. Materials and Methods

### 2.1. Plasma Collection

This study was approved by the Ethical Clearance Committee on Human Rights Related to Research Involving Human Subjects, Faculty of Medicine Ramathibodi Hospital, Mahidol University (Protocol ID 03-58-68). The Standard Operating Procedure for EDTA plasma collection created by the Early Detection Research Network (EDRN) was followed [16]. Human blood plasma samples were collected from 3 healthy volunteers (two males and one female, age 34.0±6.1 years). Blood was drawn into a 3-ml EDTA blood collection tube using a 21-gauge needle and stored at 4°C. Within 4 h after blood collection, plasma was collected by centrifugation using a swinging bucket rotor at 1,500 x g for 10 min at 4°C. The obtained plasma was aliquot and kept at -80°C until use.

### 2.2. Thermal Treatment

Three hundred microliters of plasma were transferred into a 1.5-mL polypropylene conical microcentrifuge tube (Eppendorf #022364111; Eppendorf North America, Hauppauge, NY) and incubated at 65, 75, 85, and 95°C for 20 min in Eppendorf ThermoMixer-C incubator (Eppendorf AG, Hamburg, Germany). After obtaining the optimum temperature, the incubation time was varied for 5, 10, 20, and 30 min at the fixed optimum temperature. After thermal treatment, the sample was immediately placed on ice for 5 min to allow the denatured plasma protein to aggregate and then subjected to centrifugation at 12,000 x g for 10 min. The supernatant containing TS proteins (TS soluble fraction) was collected. Protein concentration and recovery yield were estimated by the Bradford's assay. Ten micrograms of proteins in each condition were resolved on 12.5% SDS-PAGE and visualized using the blue silver CBB-G250 staining [4].

### 2.3. Immunodepletion (IMDP)

Depletion of albumin and immunoglobulin, two most abundant plasma proteins, was performed using Pierce Top2 abundant protein depletion spin column (#85161, Thermo Fisher Scientific Inc., IL, USA) according to the manufacturer's instructions. Briefly, 10 *μ*l of plasma sample was directly added to the immunodepletion spin column containing 62% slurry in 10 mM PBS, 0.15 M NaCl, 0.02% sodium azide, pH 7.4 and mixed gently. The mixture was then incubated for 30 min at room temperature with gentle end-over-end mixing every 5 min. The unbound fraction was harvested by centrifugation at 1,000 × g for 2 min and kept at -80°C until further analysis.

### 2.4. Combinatorial Peptide Ligand Libraries (CPLL)

Enrichment of low-abundance plasma proteins using the CPLL column (ProteoMiner; #163-3006, Bio-Rad Laboratories, Inc., CA, USA) was performed according to the manufacturer's instructions. Briefly, the CPLL column was prepared by adding 200 *μ*l wash buffer (BioRad) and rotating the column several times over a 5 min period. The wash buffer was removed by centrifugation at 1,000 × g for 1 min. This step was repeated once. Thereafter, 200 *μ*l of plasma was added to the column followed by incubation for 2 h at room temperature with gentle mixing. The unbound proteins were then removed by 1000 x g centrifugation for 1 min, and the column was washed twice using 200 *μ*L wash buffer (BioRad) and additionally washed by 200 *μ*L deionized water to remove unbound proteins and salt contamination. The bound proteins were eluted by adding 20 *μ*l of elution reagent (BioRad) and then incubation for 15 min with intermittent gentle mixing. The eluted proteins were collected by centrifugation at 1,000 × g for 30-60 sec. This elution step was repeated twice. The eluate was kept at -80°C until further analysis.

### 2.5. Two-Dimensional Gel Electrophoresis (2-DE) and Protein Spot Analysis

Fifty micrograms proteins from the untreated plasma and the thermal treatment conditions (3 individuals per condition) were mixed with a rehydration buffer (7 M urea, 2 M thiourea, 4% CHAPS, 0.5% (v/v) IPG buffer pH 3-10, 60 mM DTT and 40 mM Tris) and rehydrated into a 7-cm IPG strip (pH 3-10 nonlinear and/or pH 4-7 linear; GE Healthcare, Little Chalfont, UK) for 16 h at room temperature. Isoelectric focusing (IEF) was performed by the Ettan IPGphor III IEF System (GE Healthcare) at 20°C using a stepwise voltage increase to reach 9,000 Vh. The focused IPG strip was equilibrated with an equilibration buffer (6 M urea, 130 mM DTT, 112 mM Tris-HCl pH 8.8, 4% SDS, 30% glycerol and 0.002% bromophenol blue) for 15 min at room temperature with agitation, followed by another equilibration for 15 min in the same solution in which DTT was replaced by 135 mM iodoacetamide. The proteins on the equilibrated strip were separated on 12.5% SDS-PAGE using SE260 mini-Vertical Electrophoresis Unit (GE Healthcare) at 150 V for 2 h. Protein spots on the gel were visualized by blue silver CBB-G250 staining [4]. The stained gel was captured by ImageScanner III (GE Healthcare). Reproducibility of the protein spot position based on their p*I* and molecular weight was automatically detected on 2-DE by using ImageMaster 2D-Platinum software (GE Healthcare) including the protein profile pattern, the protein spot resolution, the total number of detected spots, and the normalized spot intensity [17]. Parameters used for spot detection included the minimal area of 10 pixels, the smooth factor of 2.0, and the saliency of 2.0.

### 2.6. Western Blot Analysis

Proteins (10 *μ*g/lane) were resolved on 12.5% SDS-PAGE at a constant 150V for 2 h. The separated proteins were transferred onto PVDF membranes (Immobilon-P; Millipore, MA, USA) using Trans-Blot SD semidry transfer cell (Bio-Rad). The membranes were blocked in 5% skim milk in PBS for 1 h at room temperature with agitation. After washing, the membranes were probed with antibodies against HSA (ab10241; Abcam Inc., Cambridge, MA) or IgG heavy chain (IgG HC) (P0124, DakoCytomation, Denmark) at dilution 1:1000 in 1% BSA/PBS, 4°C overnight. The membranes were washed to remove excess antibodies and then incubated in secondary antibody conjugated with HRP (at dilution 1:2000 in 1%BSA/PBS) (DakoCytomation, Denmark) at room temperature for 1 h. After washing, the membranes were incubated with enhanced chemiluminescence (ECL) (GE Healthcare), followed by detection with ImageQuant™ LAS 4000 (GE Healthcare).

### 2.7. In-Solution Tryptic Digestion

Equal protein amounts (20 *μ*g each) from 4 conditions, i.e., untreated plasma, thermal treatment, IMDP, and CPLL, were digested following modified filter-aided sample preparation (FASP) [18]. Briefly, the plasma proteins in the 3 kDa cut-off spin filter were reduced by 5 mM DTT in 8M urea/0.1 M Tris-HCl, pH 8.5 at 37°C for 1 h in the dark on Eppendorf ThermoMixer-C, subsequently concentrated by centrifugation at 14,000 rpm for 10 min. Then 100 *μ*l of 15 mM IAA in 8M urea/0.1 M Tris-HCl, pH 8.5 was added to the filter containing the reduced proteins and then incubated for 30 min at room temperature in the dark with agitation. To concentrate and discard the salt in the sample, the latter in the filter was centrifuged at 14,000 rpm for 10 min and then added with 200 *μ*L of 50 mM NH_4_HCO_3_ solution. This step was repeated twice. Proteins were digested with a final ratio of 1:50 (w/w) trypsin (Promega Corp., WI, USA) at 37°C for 16-h. The trypsin activity was stopped by adding 5% formic acid in 50% ACN and then incubated at 37°C for 20 min. The spin filter containing peptides was centrifuged at 14,000 rpm for 30 min to collect the peptides which passed through the 3 kDa cut-off filter. The peptides were dried by SpeedVac concentrator. The dried peptides were resuspended with 10 *μ*L of 0.1% formic acid.

### 2.8. Label-Free Quantitative Mass Spectrometry

Five microliters of the peptide solution were injected into Agilent 6530 Accurate-Mass Quadrupole-Time Of Flight (QTOF) mass spectrometer in standard 4GHz high resolution mode coupled to Agilent 1260 Infinity liquid chromatography with precolumn contained Zorbax 300SB-C18 (5 *μ*m, 5×0.3 mm) and analytical column contained Zorbax 300SB-C18 (3.5 *μ*m, 75 *μ*m×150 mm) using a gradient of solvent B (acetonitrile with 0.1% formic acid) in solvent A (water with 0.1% formic acid). Condition on injection was 5% solvent B and progressed to 40% solvent B for 100 min with a linear gradient and subsequently to 80% solvent B for 10 min at a flow rate of 0.5 *μ*L/min. The Agilent QTOF instrument was operated via Mass Hunter workstation data acquisition and the parameters were set as follows: MS range of 290-3000 m/z, MS/MS range of 50-1700 m/z, 20 maximum precursors per cycle, capillary voltage = 2000 V, fragmentor = 175 V, skimmer = 65 V, OCT 1 RF Vpp = 750 V, gas temperature 300°C, and drying gas 10 L/min. Raw data of all samples were searched via Spectrum Mill software against Swiss-Prot database version 20161213,* Homo sapiens* taxonomy (20,130 sequences), peptide mass tolerance ±100 ppm, MS/MS fragment mass tolerance ±0.4 Da, monoisotopic, charge 2+ to 7+, 2 missed cleavage for trypsin digestion. Peptides were identified using the score threshold >9 and the false discovery rate (FDR) <1% and quantified by the MS1-based intensity. Only peptides that presented in at least 2 out of 3 independent samples for a given condition and also pass a filter of ≥2 unique peptides per protein [19, 20] were utilized for a comparative purpose.

### 2.9. Data and Statistical Analysis

Data and statistical analysis were performed with Excel and R package MetaboanalystR [21]. The MS1 intensity of each unique peptide was normalized against the total ion intensity of its LC-MS injection. Missing values were not imputed and were set to zero by default. Expression data was preprocessed by log2 transformation and autoscaling. The self-organized heatmap was based on Pearson distance and average linkage. Venn diagram was generated by InteractiVenn [22]. A correlation matrix was plotted using Pearson correlation. Principal component analysis was performed to visualize directions of sample groups based on mass spectrometric data. Physical and chemical properties including instability index, aliphatic index, and grand average of hydropathicity (GRAVY) were computed by ProtParam tool (https://web.expasy.org/protparam). Data was presented as mean, standard error of the mean (SEM), and coefficient of variation (CV) in the independent experiments.* P* value < 0.05 was considered statistically significant.

## 3. Results and Discussion

### 3.1. The Optimum Thermal Treatment Is 95°C for 20 min

A main challenge for the optimization of thermal treatment is that differences in applied temperature and incubation time can yield various outcomes. Extreme heating or very long incubation may destroy all plasma proteins, whereas mild heating or too short incubation may not produce a stable aggregate of denatured proteins. Since the goal of this study was to apply thermal treatment to plasma proteomics, both temperature and incubation period need to be optimized to cause depletion of high abundant plasma proteins in a reproducible manner.

**Figure 1:**
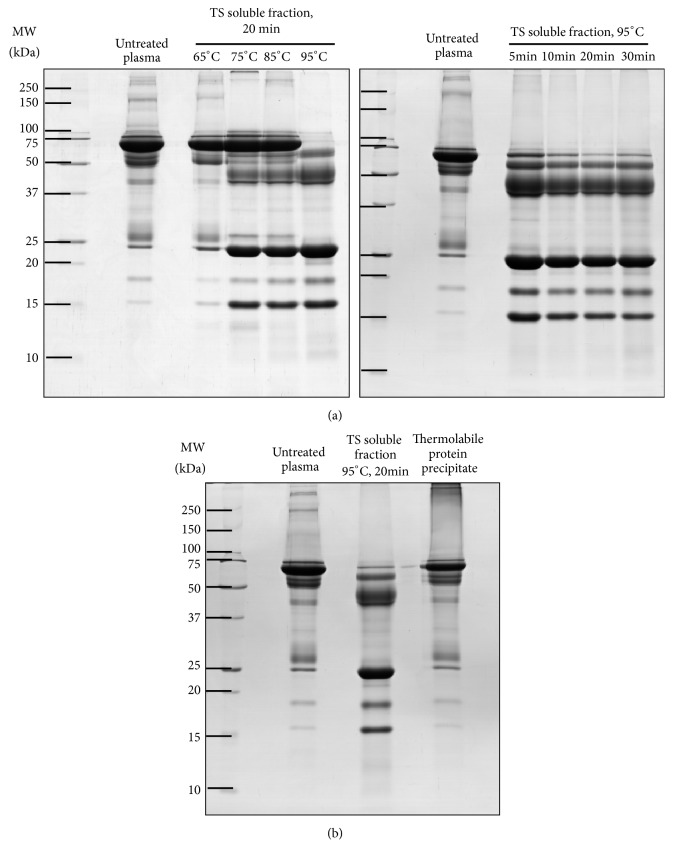
Optimization of the temperature and incubation time for thermal treatment. (a) At a fixed incubation time of 20 min, various temperatures from 65°C to 95°C affected the plasma patterns, with the prominent protein band of human serum albumin (HSA) (69 kDa) being markedly decreased at 95°C. By varying the incubation time from 5 min to 30 min at the selected temperature of 95°C, the depletion level of HSA was constant at 20 min of treatment. (b) The protein band pattern of the TS soluble fraction obtained from the optimal thermal treatment at 95°C for 20 min was compared to that of untreated plasma and the thermolabile precipitated protein fraction by 12.5% SDS-PAGE (10 *μ*g/lane).

Since the most abundant protein, HSA, constitutes over half of the proteins in plasma and can be easily detected as a 69-kDa protein band on SDS-PAGE, the optimal conditions for thermal treatment were screened by HSA depletion. The effects of different temperatures and incubation times, and optimal conditions for thermal treatment, are shown in Figure 1. For the varied temperature-fixed incubation time conditions (65 to 95°C; 20 min), the prominent band of HSA was markedly decreased at 95°C thermal treatment comparing to the other lower temperatures (Figure 1(a), left panel). For variation in incubation time (5 to 30 min) at fixed temperature (95°C), the results showed HSA depletion reached a steady state after 20-30 min (Figure 1(a), right panel).  Figure 1(b) showed that the protein band pattern of the TS soluble fraction was unique, whereas the untreated plasma and thermolabile protein precipitates showed a similar pattern. This result suggests thermal treatment extracted a thermostable subproteome from whole plasma, leaving most of the high abundant proteins, especially HSA, in the protein precipitate. Accordingly, the optimal condition for thermal treatment at 95°C, 20 min was applied for further analyses.

### 3.2. 2-DE Showed Consistency and Reproducibility of Thermostable Plasma Subproteome

**Figure 2:**
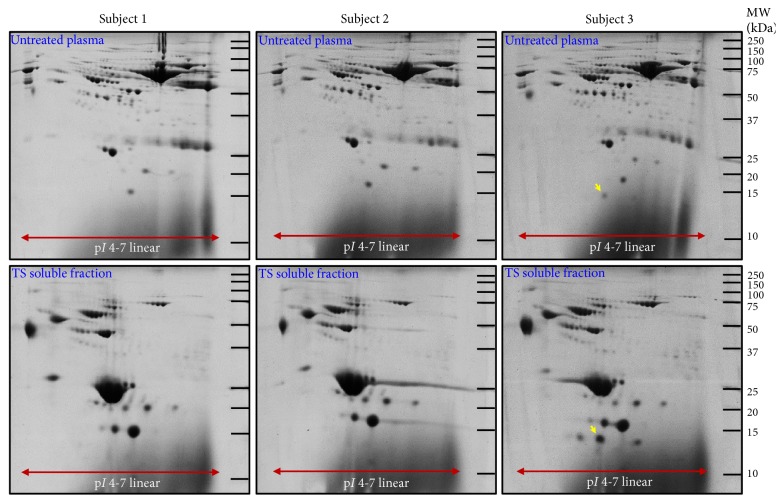
Consistency and reproducibility of thermal treatment as demonstrated by the 2-DE analysis (50 *μ*g/gel). Untreated plasma and TS soluble fraction after thermal treatment (n=3 individual samples) were separated by 2-DE using the IPG strip pH 4-7 (linear) for the first dimension and 12.5% SDS-PAGE for the second dimension. Protein spots were visualized by the blue silver CBB-G250 staining [4] and then detected by ImageMaster 2D-Platinum program. The arrow indicated a protein spot that is exclusively presented in subject 3.

The 2-DE was performed to evaluate the consistency and reproducibility of the TS soluble fraction after thermal treatment since this technique allows sensitive visualization for detecting changes in proteome profile. Plasma samples derived from 3 individuals were prepared by the optimal condition for thermal treatment. The recovery yield of thermal treatment was approximately 1% (Supplementary Table 1). This information is useful for downstream analyses, since the amount of TS protein required can be approximated from the initial plasma volume. Next, the 2-DE analysis of TS soluble fraction, as compared to the untreated plasma, is shown in Figure 2. The protein spot patterns of the untreated plasma from 3 subjects were almost identical on visual analysis, and likewise, for the results obtained from TS soluble fractions of thermal treatment. In the untreated plasma, an interindividual variation was observed in subject 3 as a faint protein spot at approximately 14-kDa molecular weight; nonetheless, the confidence level was uncertain due to low expression. After thermal treatment, this interindividual variable protein spot was clearly enriched in the corresponding TS soluble fraction of subject 3 (Figure 2), which verifies the initial observations with untreated plasma. Furthermore, the number of protein spots on the 2-DE were quantitatively counted by ImageMaster 2D-Platinum program to determine reproducibility. As a result, untreated plasma and TS soluble fractions showed intercoefficient of variations (inter-CV) of 2.3% and 4.1%, respectively (details in Supplementary Table 2). These qualitative and quantitative findings showed the consistency and reproducibility of the optimized thermal treatment and also supported further comparison with standard methods.

### 3.3. Comparison with Two Standard Methods Confirmed Applicability of Thermal Treatment

The IMDP and CPLL methods are frequently applied in plasma proteomic projects. This study, therefore, adopted Pierce-Top2 abundant protein depletion and ProteoMiner as representatives of commercially available IMDP and CPLL kits to standardize the thermal treatment. Comparison at the protein level was performed by SDS-PAGE and Western blotting for a robust evaluation of different prefractionation methods, while quantitative analysis at the peptide level was studied by label-free quantitative mass spectrometry.

**Figure 3:**
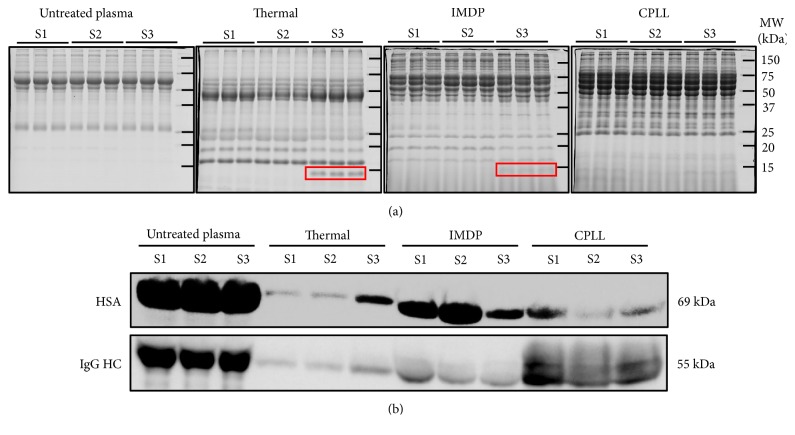
Comparison of plasma prefractionation methods using SDS-PAGE and Western blot analysis. (a) 12.5% SDS-PAGE (10 *μ*g/lane) showed consistency and reproducibility of protein band patterns with thermal treatment as well as with untreated plasma, IMDP, and CPLL. Protein bands were visualized by the blue silver CBB-G250 staining [4]. The interindividual variable proteins, so-called the Band-A, were labelled in the red square. (b) Western blot analysis (10 *μ*g/lane) demonstrated that three methods could deplete HSA and IgG, the most abundant plasma protein, as compared to the untreated plasma and thus met a required characteristic for plasma prefractionation method. Full-length immunoblots were provided in Supplementary Figure 1. Plasma samples from three subjects (S1-S3) were used for both experiments, while three technical replicates per specimen were also performed for SDS-PAGE.

Figure 3(a) demonstrated protein band patterns of the untreated plasma samples and their corresponding protein fractions after thermal treatment, IMDP, and CPLL of nine technical replicates, corresponding to three biological specimens. As expected, the protein band pattern was consistent in the same group, whereas distinct patterns were found with different prefractionation methods as compared to untreated plasma. This result suggested that thermal treatment, IMDP, and CPLL yielded different plasma subproteomes based on their mechanisms for protein isolation. The 14-kDa protein, an interindividual variation protein initially observed in Figure 2, was again detectable in thermal treatment and probably by IMDP of subject 3 as the 14-kDa protein band, the so-called “Band-A” (Figure 3(a). It was not surprising that the Band-A was not detected in the untreated plasma since the protein loading amount in SDS-PAGE (10 *μ*g/sample) was less than the 2-DE (50 *μ*g/sample). The isoelectric focusing of the 2-DE can increase the sensitivity of protein spot detection also. Presence of the Band-A in the subject 3 after IMDP supported the reliability of the thermal treatment. Further assessment was carried out by Western blot analysis.  Figure 3(b) showed the ability of various methods to deplete HSA and IgG, the two most abundant plasma proteins. Compared to the untreated plasma, three prefractionation procedures shared a common ground; even they exhibited different magnitudes of HSA and IgG depletion. Based on this promising result, thermal treatment was then further benchmarked with the IMDP and CPLL by mass spectrometric-based analysis.

After in-solution digestion, tryptic peptides of the untreated plasma, thermal treatment, IMDP, and CPLL conditions were identified and quantified by label-free quantitative mass spectrometry. Totally 963 unique peptides, corresponding to 213 unique proteins, were identified at the peptide score threshold >9 and the peptide FDR<1% (details in Supplementary Table 3). Of these, 489 unique peptides, which were present in at least 2 out of 3 samples for a given condition, and also passed a filter of ≥2 identified peptides per protein [19, 20] (details in Supplementary Table 4), were considered as having high confidence and suitable for comparison of label-free quantitative data.

**Figure 4:**
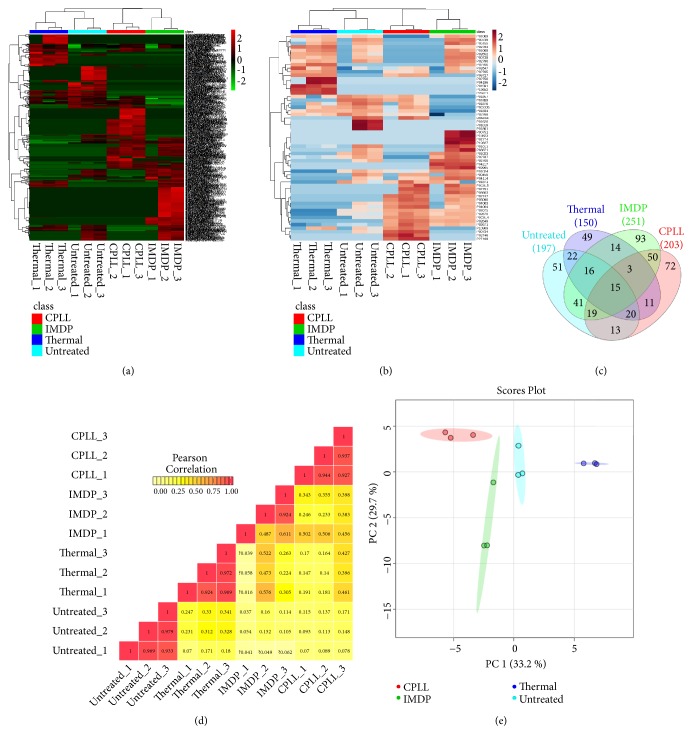
Label-free quantitative mass spectrometry with the multidimensional comparison of the untreated plasma, thermal treatment, IMDP, and CPLL. Tryptic peptides derived from three independent plasma samples of each group were identified at the peptide FDR<1% and quantified at the MS1 level by LC-QTOF. (a) The expression profiles of 489 unique peptides present in at least two out of three samples for a given prefractionation condition (details in Supplementary Table 4). (b) The heatmap of 58 unique proteins constituted from the 489 unique peptides which were found present with at least two peptides per protein (details in Supplementary Table 4). Both heatmaps at the peptide and protein levels showed significant clustering of three biological samples within the same group. (c) Venn diagram comparing the numbers of unique and shared identified peptides among four methods (details in Supplementary Table 5). (d) Pearson correlation of 12 samples based on their expression profiles. Numbers in the correlation matrix represent the correlation coefficient (*r*), where* r*=1 is a perfect relationship and* r*=0 shows no association between samples. (e) The unsupervised principal component analysis indicating that each prefractionation method produced unique sets of peptide components. These multidimensional data suggested that each prefractionation method provided a distinct plasma subproteome, which did not replace but was instead complementary to each other. The detailed information regarding all identified peptides, sequences, scores, FDR, and relative intensities are available in Supplementary Table 3. Abbreviations: FDR, false discovery rate; LC-QTOF, liquid chromatography coupled to Quadrupole-Time Of Flight.

A multidimensional comparison was then performed using a data-driven approach. Relative intensities of 489 unique peptides (Figure 4(a)) and their corresponding 58 unique proteins (Figure 4(b)) were present in the self-clustered heatmaps. Expression profiles with unsupervised clustering clearly distinguished untreated plasma, thermal treatment, IMDP, and CPLL, consistent with the previous finding (Figure 3(a)). The Venn diagram demonstrated that only 15 peptides were shared among all groups, whereas up to 49-93 peptides were uniquely present with each prefractionation method (Figure 4(c)). Next, the expression profiles of three biological samples within the same group showed a good correlation (Figure 4(d)), while the correlation coefficients were very low between different methods. Furthermore, the principal component analysis revealed four distinct directions belonging to the untreated plasma, thermal treatment, IMDP, and CPLL datasets (Figure 4(e)). In addition, the number of peptide spectrum matches (#PSMs) of serum albumin and immunoglobulins (Supplementary Figure 2) resembled Western blot analysis as shown in Figure 3(b). Moreover, physical and chemical properties of 44 identified proteins between untreated plasma and thermal treatment were compared by computation of molecular weight (MW), isoelectric point (p*I*), instability index, aliphatic index, and grand average of hydropathicity (GRAVY). Note that the aliphatic index is a positive factor for the increase of thermostability of globular proteins [23]. The prediction value of protein properties showed that the proteins in thermal treatment condition had lower molecular weight, lower p*I*, lower instability index, higher aliphatic index, and higher GRAVY index than the untreated plasma proteins (Supplementary Figure 3 and Supplementary Table 6). This computational metrics convey a clear effect of the thermal treatment on various changes in the physicochemical properties of the proteins. Taken together, this multidimensional comparison supported the conclusion that the thermal treatment yielded a distinct plasma subproteome which did not replace but was instead complementary to the IMDP and CPLL.

**Figure 5:**
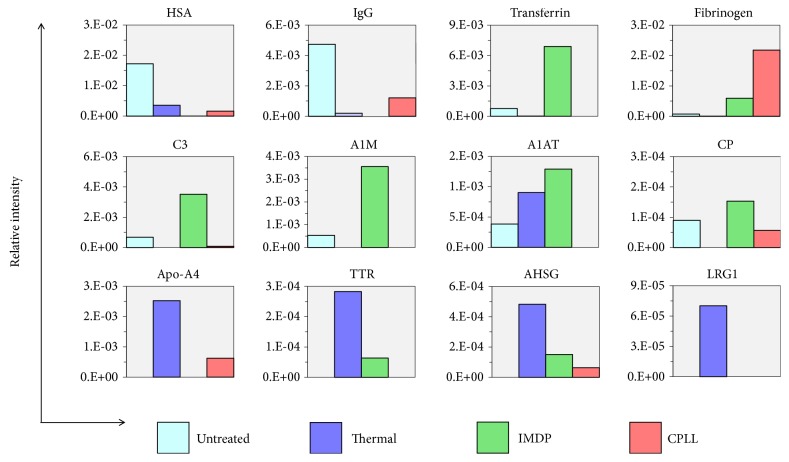
Expression data of 12 selected proteins among various plasma prefractionation methods. Bar plot represented the average protein intensity. Abbreviation: HSA, human serum albumin; IgG, immunoglobulin G; C3, complement C3; A1M, alpha-1 microglobulin; A1AT, alpha-1 antitrypsin; CP, ceruloplasmin; Apo-A4, apolipoprotein A4; TTR, transthyretin; AHSG, alpha-2-HS-glycoprotein; LRG1, leucine-rich alpha-2 glycoprotein 1.

From a practical standpoint, plasma proteomic studies commonly interpret findings at the protein level. To gain insight into the applicability of thermal treatment, 12 proteins were selected with relative abundance ranging from the highest (HSA and IgG), high (transferrin, fibrinogen, complement C3, alpha-1 microglobulin (A1M), alpha-1 antitrypsin (A1AT), ceruloplasmin (CP)) to intermediate-low abundance (apolipoprotein A4 (Apo-A4), transthyretin (TTR), alpha-2-HS-glycoprotein (AHSG) and leucine-rich alpha-2 glycoprotein 1 (LRG1)) as shown in Figure 5. As expected, HSA and IgG were depleted by the thermal treatment, IMDP, and CPLL as compared to the untreated plasma. Inflammatory-associated markers [24–28], i.e., transferrin, fibrinogen, complement C3, A1M, A1AT, and CP, were enriched at various magnitudes by IMDP and CPLL, but were mostly depleted by the thermal treatment. Apo-A4, TTR, AHSG, and LRG1 which were previously reported as the markers associated with ovarian tumor [29], gastric cancer [30], colorectal cancer [31], and cholangiocarcinoma [32], respectively, were highly enriched by the thermal treatment. These data suggest the potential applicability of thermal treatment as a plasma prefractionation method for studying cancer biomarkers, providing higher signals with cancer-associated markers and lower noise with HSA, IgG, and inflammatory-associated proteins.

### 3.4. Thermal Treatment Has a Strategic Benefit for Large-Scale Plasma Proteomic Studies

In the end, the selection of prefractionation method should consider the specific need of each project [3]. There is no perfect method since different approaches have unique characteristics with both benefits and disadvantages. From this point of view, procedure characteristics of thermal treatment, IMDP and CPLL are listed in the following section and discussed regarding the sample loading capacity and recovery yield, the complexity of procedure, and the unit cost. The source information of IMDP and CPLL was based on the kit instructions and the vendor websites as last checked on January 5, 2019.

The sample loading capacity and recovery yield of prefractionation method would affect the design of downstream analyses. In this regard, thermal treatment, IMDP, and CPLL had the sample loading capacity of 300 *μ*L plasma (~20g protein), 10 *μ*L plasma (~0.8g), and ≥0.01g protein, respectively, with their corresponding recovery yields of 1%, 10.0%, and 1.3%. The recovery yields of IMDP and CPLL in this study were also consistent with a previous report (10.9% and 1.1%, respectively) [33], indicating the reproducibility of the commercial methods. IMDP exhibited a higher recovery yield than others, but this is probably due to the saturation of immunoaffinity beads [33]. Considering a scaling up, thermal treatment has apparently no limit of sample loading capacity, even though the starting plasma volume of 300-1000 *μ*L should return a sufficient protein amount (200-650 mg) for most downstream analyses. Also, this scaling up is associated with a minimal cost burden since no specific material is required for thermal treatment.

The complexity of procedure could reduce the sample throughput and productivity. This characteristic could be objectively measured by the step of procedure and the working time [34]. Thermal treatment and IMDP consist of two main steps (sample loading and centrifugation), whereas CPLL needs four (column pretreatment, sample loading, centrifugation, and protein elution). The working time of thermal treatment and IMDP is also similar (35 min and 45 min, respectively), while CPLL requires at least 150 min to complete the process.

The unit cost of prefractionation method could be a burden for large-scale projects. In our setting, thermal treatment has an actual cost of 0.1 USD/sample (as estimated by the cost of electricity for the heating process). The unit cost of Pierce top 2 abundant protein depletion spin column (the IMDP representative) is 30.6 USD/sample (184 USD per 6 columns; #85161, www.thermofisher.com) and that of ProteoMiner Protein Enrichment Small-Capacity (the CPLL representative) is 72.7 USD/sample (727 USD per 10 columns; #1633006, www.bio-rad.com). Although this information cannot cover all prefractionation kits available in the market, it is undoubted that thermal treatment is cost-effective and has less workload than the comparators. Accordingly, it is attractive to apply the thermal treatment in plasma proteomic studies involving large populations and multicenter cohorts in the future.

## 4. Conclusions

In conclusion, this study demonstrated that the optimized and standardized thermal treatment is a reliable and reproducible plasma prefractionation for proteomic analysis. Information on its potential application supported the role of thermal treatment in large-scale proteomic studies involving biomarker validation and cost-effective proteomic biomarker assays.

## Data Availability

The proteomic data used to support the findings of this study were included within the Supplementary Information.

## Supplementary Materials

Supplementary 1
* Supplementary Table 1*: recovery yield of plasma prefractionation by thermal treatment.* Supplementary Table 2*: coefficient of variation (CV) found with the numbers of the detected protein spots on 2-DE gels (n=3 subjects).* Supplementary Table 3*: total of 963 unique peptides identified at the peptide score>9 and the peptide FDR<1% by LC-QTOF.* Supplementary Table 4*: detailed information on 489 unique peptides corresponding to 58 unique proteins for comparative purposes.* Supplementary Table 5*: detailed information regarding the unique peptides present in each compartment of Venn diagram.* Supplementary Table 6:* prediction of physical and chemical properties of 44 identified proteins in untreated and thermal conditions by ProtParam tool (https://web.expasy.org/protparam).

Supplementary 2
* Supplementary Figure 1:* full-length immunoblot results of HSA (69 kDa) and IgG HC (55 kDa) corresponding to a group of cropped blot images shown in Figure 3(b).* Supplementary Figure 2:* number of peptide spectrum matches (#PSMs) of serum albumin and immunoglobulins by LC-QTOF. Abbreviations: CPLL, combinatorial peptide ligand libraries; IMDP, immunodepletion; LC, liquid chromatography; QTOF, Quadrupole-Time Of Flight.* Supplementary Figure 3:* Venn diagram demonstrating the numbers of identified proteins in untreated and thermally treated plasma and bar graphs showing their predictive physical and chemical properties including molecular weight (MW), isoelectric point (p*I*), instability index, aliphatic index, and grand average of hydropathicity (GRAVY) index. A group of common proteins shared in both conditions was labelled by black color.

## References

[1] Anderson N. L., Anderson N. G. (2002). The human plasma proteome: history, character, and diagnostic prospects. *Molecular & Cellular Proteomics*.

[2] Taguchi A., Hanash S. M. (2013). Unleashing the power of proteomics to develop blood-based cancer markers. *Clinical Chemistry*.

[3] Chutipongtanate S., Chatchen S., Svasti J. (2017). Plasma prefractionation methods for proteomic analysis and perspectives in clinical applications. *Proteomics - Clinical Applications*.

[4] Candiano G., Bruschi M., Musante L. (2004). Blue silver: a very sensitive colloidal coomassie G-250 staining for proteome analysis. *Electrophoresis*.

[5] Kawaguchi S., Kuramitsu S. (1995). Separation of heat-stable proteins from Thermus thermophilus HB8 by two-dimensional electrophoresis. *Electrophoresis*.

[6] Nawata S., Suminami Y., Hirakawa H. (2001). Electrophoretic characterization of heat-stable squamous cell carcinoma antigen. *Electrophoresis*.

[7] Kwon S., Jung Y., Lim D. (2008). Proteomic analysis of heat-stable proteins in Escherichia coli. *BMB Reports*.

[8] Robinson A. A., Westbrook J. A., English J. A., Borén M., Dunn M. J. (2009). Assessing the use of thermal treatment to preserve the intact proteomes of post-mortem heart and brain tissue. *Proteomics*.

[9] Ofori J. A., Hsieh Y.-H. P. (2015). Characterization of a 60-kDa thermally stable antigenic protein as a marker for the immunodetection of bovine plasma-derived food ingredients. *Journal of Food Science*.

[10] Csobán Z., Kállai-Szabó B., Kállai-Szabó N., Sebe I., Gordon P., Antal I. (2015). Improvement of mechanical properties of pellet containing tablets by thermal treatment. *International Journal of Pharmaceutics*.

[11] Moshkovskii S. A., Serebryakova M. V., Kuteykin-Teplyakov K. B. (2005). Ovarian cancer marker of 11.7 kDa detected by proteomics is a serum amyloid A1. *Proteomics*.

[12] Goufman E. I., Moshkovskii S. A., Tikhonova O. V. (2006). Two-dimensional electrophoretic proteome study of serum thermostable fraction from patients with various tumor conditions. *Biochemistry (Moscow)*.

[13] Saguer E., Alvarez P., Ismail A. A. (2012). Heat-induced denaturation/aggregation of porcine plasma and its fractions studied by FTIR spectroscopy. *Food Hydrocolloids*.

[14] Saguer E., Alvarez P., Fort N. (2015). Heat-Induced gelation mechanism of blood plasma modulated by cysteine. *Journal of Food Science*.

[15] Dàvila E., Parés D., Howell N. K. (2006). Fourier transform Raman spectroscopy study of heat-induced gelation of plasma proteins as influenced by pH. *Journal of Agricultural and Food Chemistry*.

[16] Tuck M. K., Chan D. W., Chia D. (2009). Standard operating procedures for serum and plasma collection: early detection research network consensus statement standard operating procedure integration working group. *Journal of Proteome Research*.

[17] Magdeldin S., Enany S., Yoshida Y. (2014). Basics and recent advances of two dimensional- polyacrylamide gel electrophoresis. *Clinical Proteomics*.

[18] Wiśniewski J. R., Zougman A., Nagaraj N., Mann M. (2009). Universal sample preparation method for proteome analysis. *Nature Methods*.

[19] Higgs R. E., Knierman M. D., Gelfanova V., Butler J. P., Hale J. E. (2005). Comprehensive label-free method for the relative quantification of proteins from biological samples. *Journal of Proteome Research*.

[20] Wang M., You J., Bemis K. G., Tegeler T. J., Brown D. P. G. (2008). Label-free mass spectrometry-based protein quantification technologies in proteomic analysis. *Briefings in Functional Genomics & Proteomics*.

[21] Chong J., Soufan O., Li C. (2018). MetaboAnalyst 4.0: towards more transparent and integrative metabolomics analysis. *Nucleic Acids Research*.

[22] Heberle H., Meirelles V. G., da Silva F. R., Telles G. P., Minghim R. (2015). InteractiVenn: a web-based tool for the analysis of sets through Venn diagrams. *BMC Bioinformatics*.

[23] Ikai A. (1980). Thermostability and aliphatic index of globular proteins. *The Journal of Biochemistry*.

[24] Baune B. T., Neuhauser H., Ellert U., Berger K. (2010). The role of the inflammatory markers ferritin, transferrin and fibrinogen in the relationship between major depression and cardiovascular disorders - the German health interview and examination survey. *Acta Psychiatrica Scandinavica*.

[25] Shields K. J., Mollnes T. E., Eidet J. R. (2017). Plasma complement and vascular complement deposition in patients with coronary artery disease with and without inflammatory rheumatic diseases. *PLoS ONE*.

[26] Vyssoulis G. P., Tousoulis D., Antoniades C., Dimitrakopoulos S., Zervoudaki A., Stefanadis C. (2007). Alpha-1 microglobulin as a new inflammatory marker in newly diagnosed hypertensive patients. *American Journal of Hypertension*.

[27] Ottaviani S., Gorrini M., Scabini R. (2011). C reactive protein and alpha1-antitrypsin: relationship between levels and gene variants. *Translational Research*.

[28] Gitlin J. D. (1988). Transcriptional regulation of ceruloplasmin gene expression during inflammation. *The Journal of Biological Chemistry*.

[29] Fan N., Kang R., Ge X. (2014). Identification alpha-2-HS-glycoprotein precursor and tubulin beta chain as serology diagnosis biomarker of colorectal cancer. *Diagnostic Pathology*.

[30] Shimura T., Shibata M., Gonda K. (2018). Serum transthyretin level is associated with prognosis of patients with gastric cancer. *Journal of Surgical Research*.

[31] Dieplinger H., Ankerst D. P., Burges A. (2009). Afamin and apolipoprotein A-IV: novel protein markers for ovarian cancer. *Cancer Epidemiology, Biomarkers & Prevention*.

[32] Sandanayake N. S., Sinclair J., Andreola F. (2011). A combination of serum leucine-rich alpha-2-glycoprotein 1, CA19-9 and interleukin-6 differentiate biliary tract cancer from benign biliary strictures. *British Journal of Cancer*.

[33] Pisanu S., Biosa G., Carcangiu L., Uzzau S., Pagnozzi D. (2018). Comparative evaluation of seven commercial products for human serum enrichment/depletion by shotgun proteomics. *Talanta*.

[34] Chutipongtanate S., Changtong C., Weeraphan C., Hongeng S., Srisomsap C., Svasti J. (2015). Syringe-push membrane absorption as a simple rapid method of urine preparation for clinical proteomics. *Clinical Proteomics*.

