# Supplementary material for: Optimization and Standardization of Thermal Treatment as a Plasma Prefractionation Method for Proteomic Analysis
Source: Biomed Res Int. 2019 Apr 30;2019:8646039. doi: 10.1155/2019/8646039 (PMC6515177; doi:10.1155/2019/8646039)
Supplement: Supplementary 1 — Supplementary Table 1: recovery yield of plasma prefractionation by thermal treatment. Supplementary Table 2: coefficient of variation (CV) found with the numbers of the detected protein spots on 2-DE gels (n=3 subjects). Supplementary Table 3: total of 963 unique peptides identified at the peptide score>9 and the peptide FDR<1% by LC-QTOF. Supplementary Table 4: detailed information on 489 unique peptides corresponding to 58 unique proteins for comparative purposes. Supplementary Table 5: detailed information regarding the unique peptides present in each compartment of Venn diagram. Supplementary Table 6: prediction of physical and chemical properties of 44 identified proteins in untreated and thermal conditions by ProtParam tool (https://web.expasy.org/protparam). [file 8646039.f1.pptx]

## Slide 1
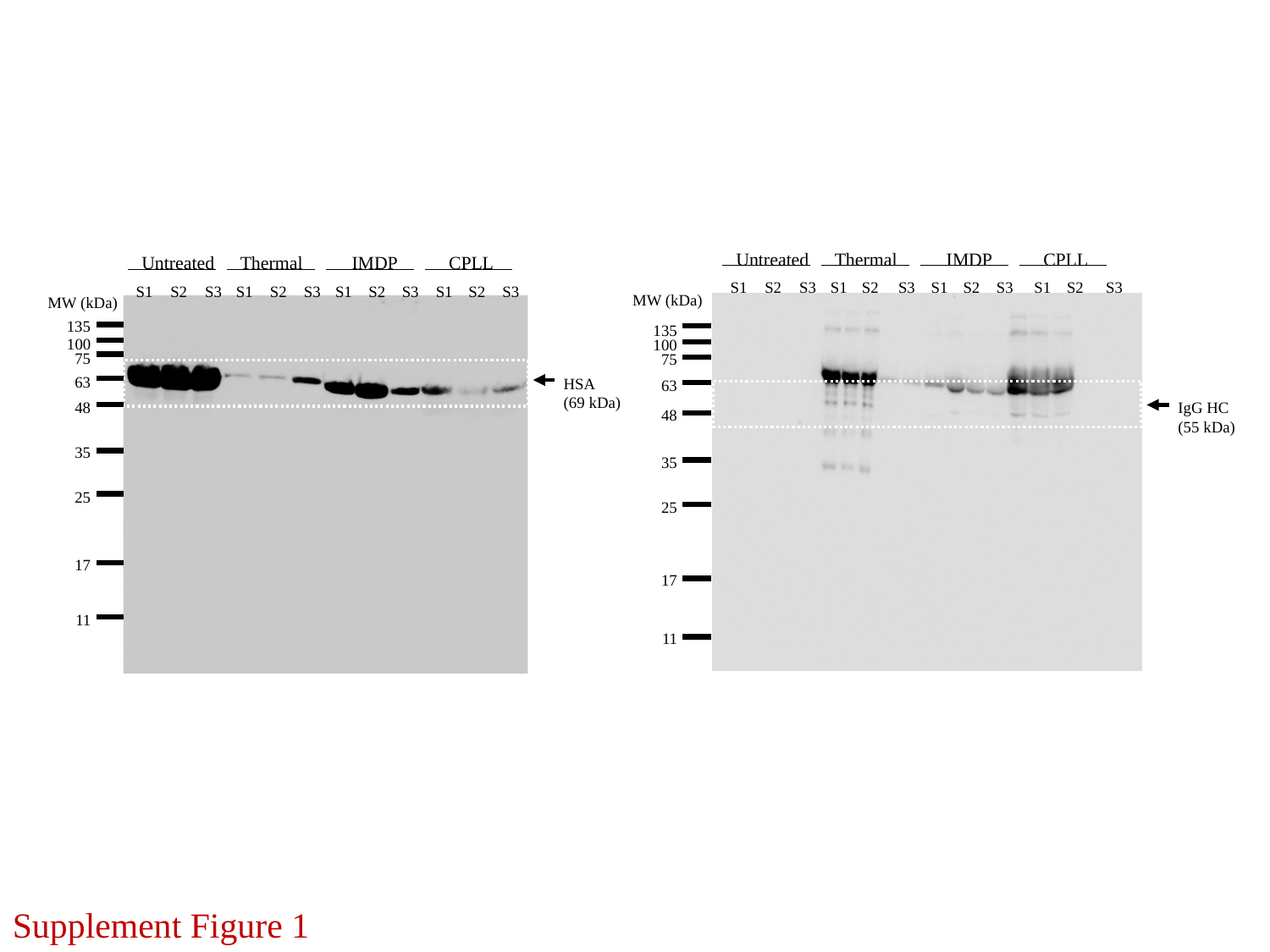

Untreated
Thermal
IMDP
CPLL
S1
S2
S3
S1
S2
S3
S1
S2
S3
S1
S2
S3
Untreated
Thermal
IMDP
CPLL
S1
S2
S3
S1
S2
S3
S1
S2
S3
S1
S2
S3
MW (kDa)
MW (kDa)
135
100
75
63
48
35
25
17
11
135
100
75
HSA
(69 kDa)
63
IgG HC
(55 kDa)
48
35
25
17
11
Supplement Figure 1

## Slide 2
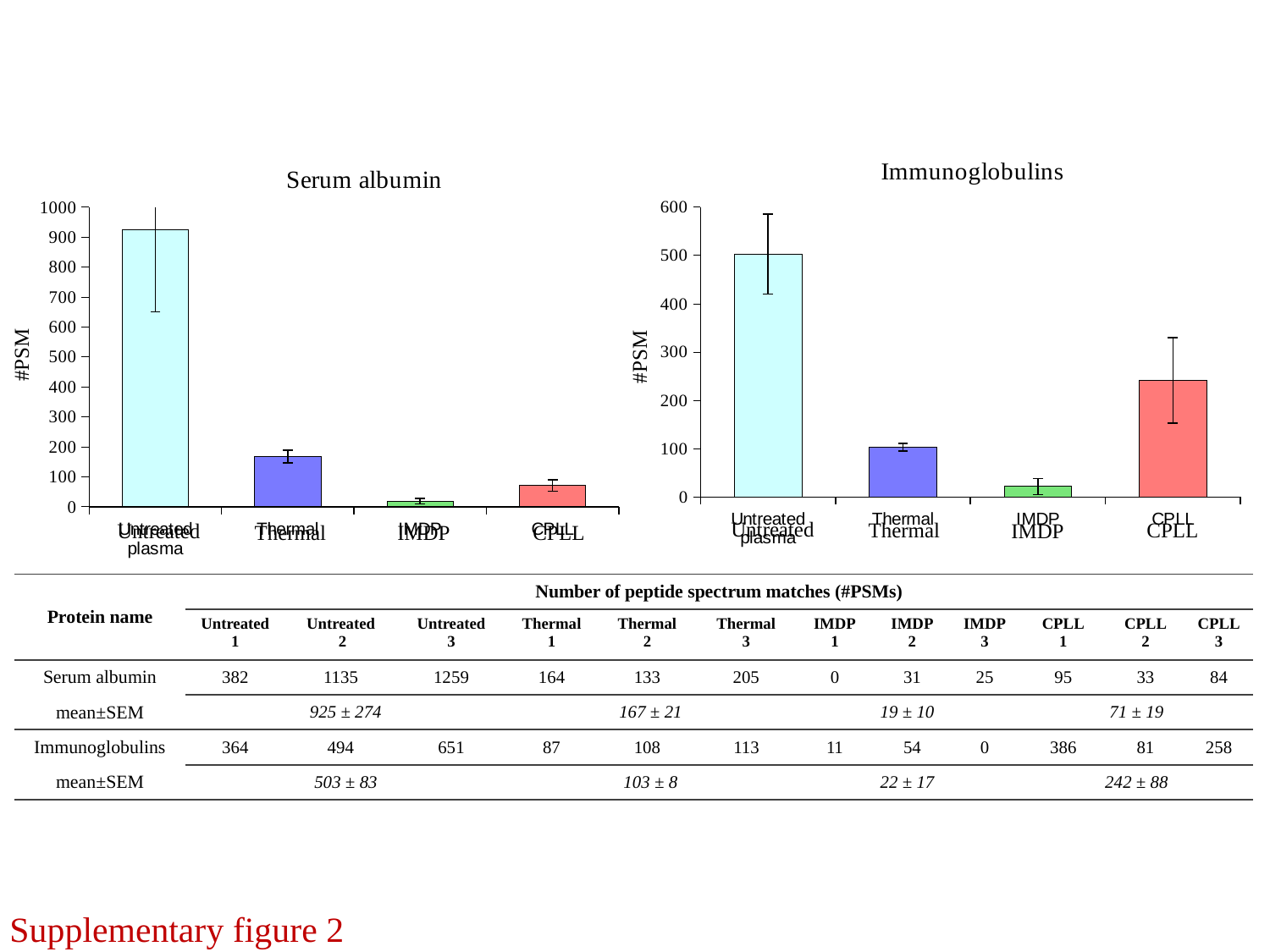

### Chart: Serum albumin
| Category | |
|---|---|
| Untreated plasma | 925.333333333333 |
| Thermal | 167.333333333333 |
| IMDP | 18.6666666666667 |
| CPLL | 70.6666666666667 |
### Chart: Immunoglobulins
| Category | |
|---|---|
| Untreated plasma | 503.0 |
| Thermal | 102.666666666667 |
| IMDP | 21.6666666666667 |
| CPLL | 241.666666666667 |#PSM
#PSM
Untreated
CPLL
Thermal
IMDP
Untreated
CPLL
Thermal
IMDP
| Protein name | Number of peptide spectrum matches (#PSMs) | | | | | | | | | | | |
| --- | --- | --- | --- | --- | --- | --- | --- | --- | --- | --- | --- | --- |
| | Untreated 1 | Untreated 2 | Untreated 3 | Thermal 1 | Thermal 2 | Thermal 3 | IMDP 1 | IMDP 2 | IMDP 3 | CPLL 1 | CPLL 2 | CPLL 3 |
| Serum albumin | 382 | 1135 | 1259 | 164 | 133 | 205 | 0 | 31 | 25 | 95 | 33 | 84 |
| mean±SEM | 925 ± 274 | | | 167 ± 21 | | | 19 ± 10 | | | 71 ± 19 | | |
| Immunoglobulins | 364 | 494 | 651 | 87 | 108 | 113 | 11 | 54 | 0 | 386 | 81 | 258 |
| mean±SEM | 503 ± 83 | | | 103 ± 8 | | | 22 ± 17 | | | 242 ± 88 | | |
Supplementary figure 2

## Slide 3
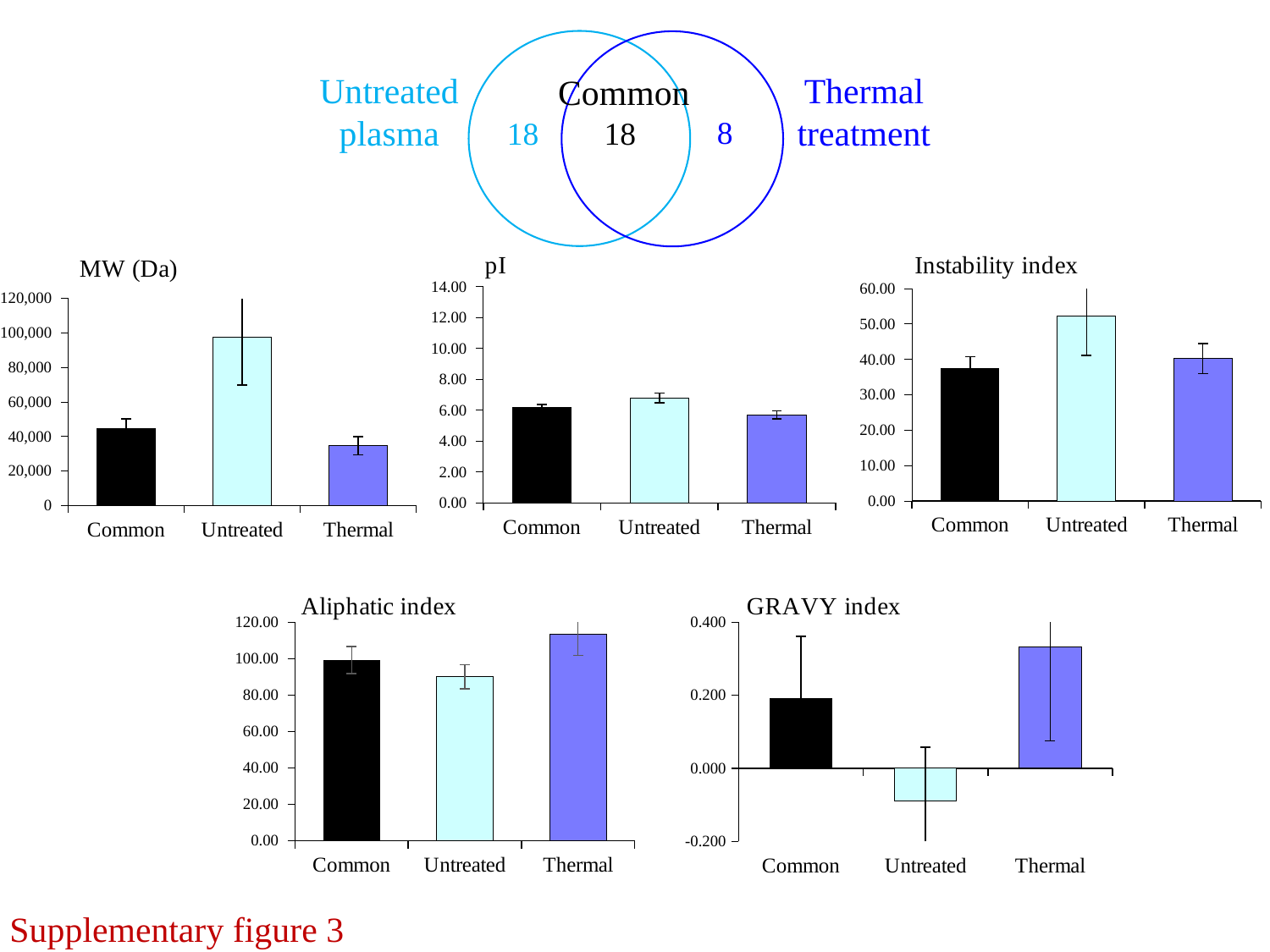

Untreated
plasma
Thermal
treatment
8
18
18
### Chart:
| Category | Instability index |
|---|---|
| Common | 37.4639473684211 |
| Untreated | 52.3 |
| Thermal | 40.19 |
### Chart: pI
| Category | pI |
|---|---|
| Common | 6.17111111111111 |
| Untreated | 6.81 |
| Thermal | 5.71 |
### Chart: MW (Da)
| Category | MW |
|---|---|
| Common | 44704.35 |
| Untreated | 97777.1 |
| Thermal | 34675.3 |
### Chart:
| Category | GRAVY index |
|---|---|
| Common | 0.192447368421053 |
| Untreated | -0.089 |
| Thermal | 0.333 |
### Chart:
| Category | Aliphatic index |
|---|---|
| Common | 99.2918421052631 |
| Untreated | 90.01 |
| Thermal | 113.43 |Common
Supplementary figure 3
